# Functional and Structural Changes in the Inner Ear and Cochlear Hair Cell Loss Induced by Hypergravity

**DOI:** 10.3390/ijms26020758

**Published:** 2025-01-17

**Authors:** Jin Sil Choi, Kyu-Sung Kim, Hyun Ji Kim

**Affiliations:** 1 Department of Otorhinolaryngology-Head and Neck Surgery, College of Medicine, Inha University, Incheon 22332, Republic of Korea; true_choi@inha.ac.kr (J.S.C.); stedman@inha.ac.kr (K.-S.K.); 2 Inha Research Institute for Aerospace Medicine, College of Medicine, Inha University, Incheon 22332, Republic of Korea

**Keywords:** hypergravity, inner ear, cochlear hair cell, synapse plasticity

## Abstract

Gravitational changes have been shown to cause significant abnormalities in various body systems, including the cardiovascular, immune, vestibular, and musculoskeletal systems. While numerous studies have examined the response of the vestibular system to gravitational stimulation, research on functional changes in the peripheral inner ear remains limited. The inner ear comprises two closely related structures: the vestibule and cochlea. These components share similar structures and neural functions, highlighting the importance of investigating changes in auditory nerve cells in response to gravitational alterations. To address this gap, we studied the functional and structural changes in the inner ear following exposure to hypergravity stimuli. Our findings demonstrate changes in auditory brainstem responses (ABRs) in the cochlea. ABR recordings were used to analyze click thresholds, as well as the amplitude and latency of tone bursts. The click thresholds at all frequencies increased in the group exposed to hypergravity in the long term. Additionally, tone burst results revealed significantly reduced amplitudes at high frequencies and delayed latencies in the hypergravity models. Notably, greater hair cell loss was observed in the middle and basal turns of the cochlea, indicating that mid and high-frequency regions are more vulnerable to hypergravity stimulation. Furthermore, nerve damage on the cochlear surface was evident in subjects exposed to 4G stimulation for 4 weeks. These findings suggest that the inner ear and its neural activity can be functionally and structurally affected by prolonged exposure to hypergravity.

## 1. Introduction

The effects of gravitational changes on skeletal, muscular, cardiovascular, immune, and vestibular systems have been well-documented [1]. The vestibular system plays a crucial role in sensing and adapting to gravity [2,3], leading to extensive research on how it responds to gravitational changes [2,4,5,6]. However, the effects of altered gravity on the auditory system, which shares functional and structural similarities with the vestibular system, remain insufficiently studied. Recent advancements in space exploration and aviation technology have increased human exposure to gravitational changes [7], raising concerns about their potential impact on auditory function [8].

The effects of hypergravitational stimulation on hearing may occur through several mechanisms. First, it can induce physical stress on inner ear structures, particularly the cochlea [9]. Second, it may alter blood flow to the inner ear, affecting the supply of oxygen and nutrients to hearing cells [8,10]. Third, gravitational changes can affect cellular structures, connectivity, and functional signal transmission in the vestibular system, potentially influencing inner ear sensory functions and hearing [2,10]. Hearing loss, characterized by impaired sound perception [11], has been linked to poor balance [12,13], underscoring the intricate relationship between hearing and balance. This study explores the effects of hypergravitational stimulation on hearing loss.

We investigated patterns of hearing changes, the underlying neurological mechanisms, and the effects on inner ear structure and function in animal models exposed to 4G hypergravity stimulation for 24 h, 2 weeks, and 4 weeks. We evaluated the effects of hypergravity stimulation on the auditory system using auditory brainstem responses (ABRs) and histological analyses.

To confirm the close association between the presynaptic ribbon and the postsynaptic receptor complex, we quantified and analyzed structural synaptic plasticity using immunostaining methods. Our aim was to provide a broad perspective on synaptic plasticity alterations and maintenance following long-term hypergravity stimuli exposure. The results of this study will deepen our understanding of the effects of hypergravity on the auditory system and provide important baseline medical preparations for future space exploration.

## 2. Results

### 2.1. ABR Recordings

**Figure 1:**
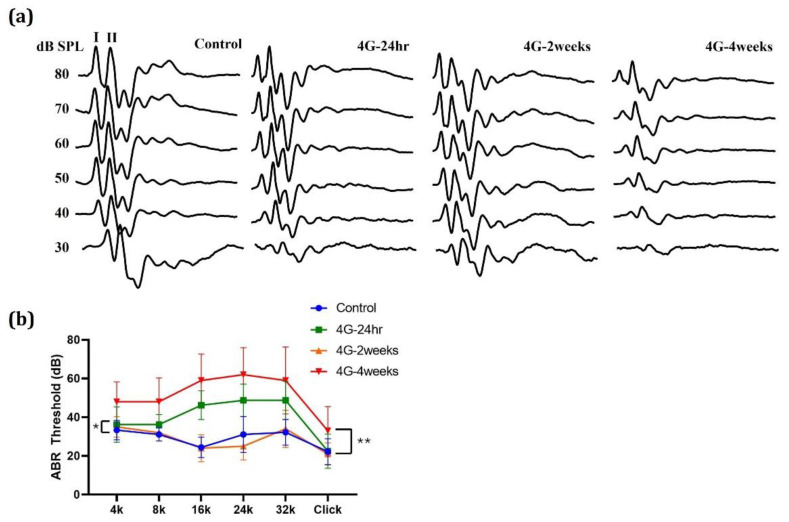
Changes in auditory brainstem response (ABR) thresholds under hypergravity stimulation. (**a**) ABRs to clicks were recorded at 24 h, 2 weeks, and 4 weeks after hypergravity exposure. (**b**) The hearing threshold was found to be slightly increased under the hypergravity (4G) condition. Control vs. 4G-24hr; * (*p* = 0.0332), control vs. 4G-2weeks; not significantly different, control vs. 4G-4weeks; ** (*p* = 0.0023).

Using a hypergravity device, a gravity stimulation animal model was established by exposing animals to 4G stimulation for 24 h, 2 weeks, and 4 weeks. ABRs were analyzed at the end of hypergravity stimulation. Analyses of hearing thresholds in response to clicking and pure-tone chirps (4, 8, 16, 24, and 32 kHz) showed a significant increase in sound levels (Figure 1). In the case of click threshold, compared to the control group, hearing loss of approximately 10 dB (control group: Mean 22.222 ± 6.667 dB, 4G-4weeks group: Mean 33.000 ± 12.517 dB) was observed in the group exposed to hypergravity for 4 weeks, which was statistically significant (** *p* = 0.0023). At the tone burst, the threshold was increased by about 15 dB (control group: Mean 33.333 ± 5.000 dB, 4G-4weeks group: Mean 48.000 ± 10.328 dB) at 4 kHz, about 16 dB (control group: Mean 31.111 ± 5.000 dB) at 8 kHz. Additionally, the threshold was increased by about 30 dB at high frequencies (control group: Mean 24.444 ± 5.270 dB, 4G-4weeks group: Mean 59.000 ± 13.703 dB at 16 kHz, control group: Mean 31.111 ± 9.280 dB, 4G-4weeks group: Mean 62.000 ± 13.984 dB at 24 kHz, and control group: Mean 32.222 ± 6.667 dB, 4G-4weeks group: Mean 59.000 ± 17.288 dB at 32 kHz).

**Figure 2:**
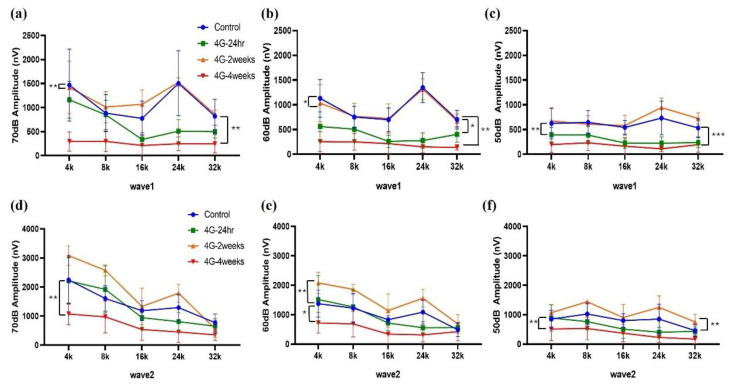
Assessment of auditory function in hypergravity models. This figure shows auditory function assessment in hypergravity models via auditory brainstem response (ABR) amplitude analysis. (**a**–**c**) Wave 1 analysis: These panels show the amplitude changes in wave 1 at 70, 60, and 50 dB across experimental groups. (**d**–**f**) Wave 2 amplitude analysis: ABR amplitudes were analyzed in 70 dB to 50 dB at low, mid, and high frequencies. The amplitude decreased in the 4G-4weeks group compared to that in the control group in both wave 1 and wave 2. * *p* < 0.05, ** *p* < 0.01, and *** *p* < 0.001.

**Figure 3:**
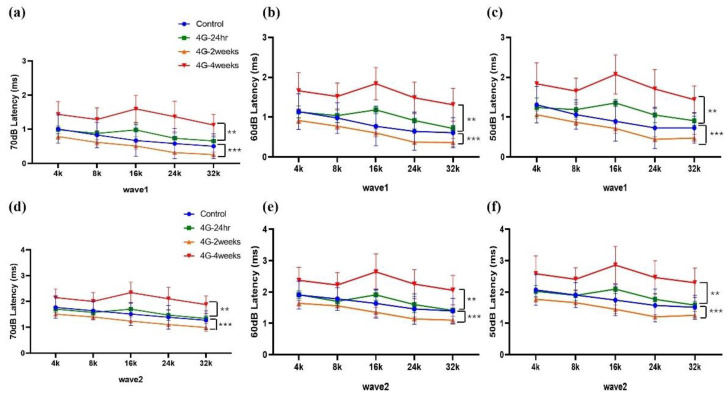
ABR latency changes in wave 1 and wave 2 in hypergravity models. ABR latency was analyzed to represent the time interval between the onset of auditory stimulation and the occurrence of a specific wave in the ABR waveform. (**a**–**c**) Wave 1 latency analysis: Changes in latency at 4, 8, 16, 24, and 32 kHz from 70 dB to 50 dB were analyzed. (**d**–**f**) Wave 2 latency analysis: The latency changed in wave 2 from 70 dB to 50 dB in the control and experimental models. The latency was delayed in the 4G-4weeks group compared to that in the control group in both wave 1 and wave 2. ** *p* < 0.01, and *** *p* < 0.001.

Changes in amplitude and latency according to frequency were analyzed for wave 1 and wave 2. At wave 1 of 70 dB, 60 dB, and 50 dB, the amplitude was significantly reduced in the 4G-4weeks group compared to the control group (Figure 2a–c), and latency was delayed (Figure 3a–c). The amplitude decreased from 70 to 50 dB in wave 2, with the latency delayed (Figure 3d,e). In pure-tone pips, hearing damage was observed in the 4G-4weeks group compared to the control group at all frequencies. In particular, distinct hearing changes were observed in wave 1 and wave 2 at high frequencies. This suggests that prolonged exposure to hypergravity stimulation affects hearing loss and that high-frequency subjects are more sensitive to gravitational stimulation than low-frequency subjects.

### 2.2. Immunostaining of Cochlear Hair Cells

**Figure 4:**
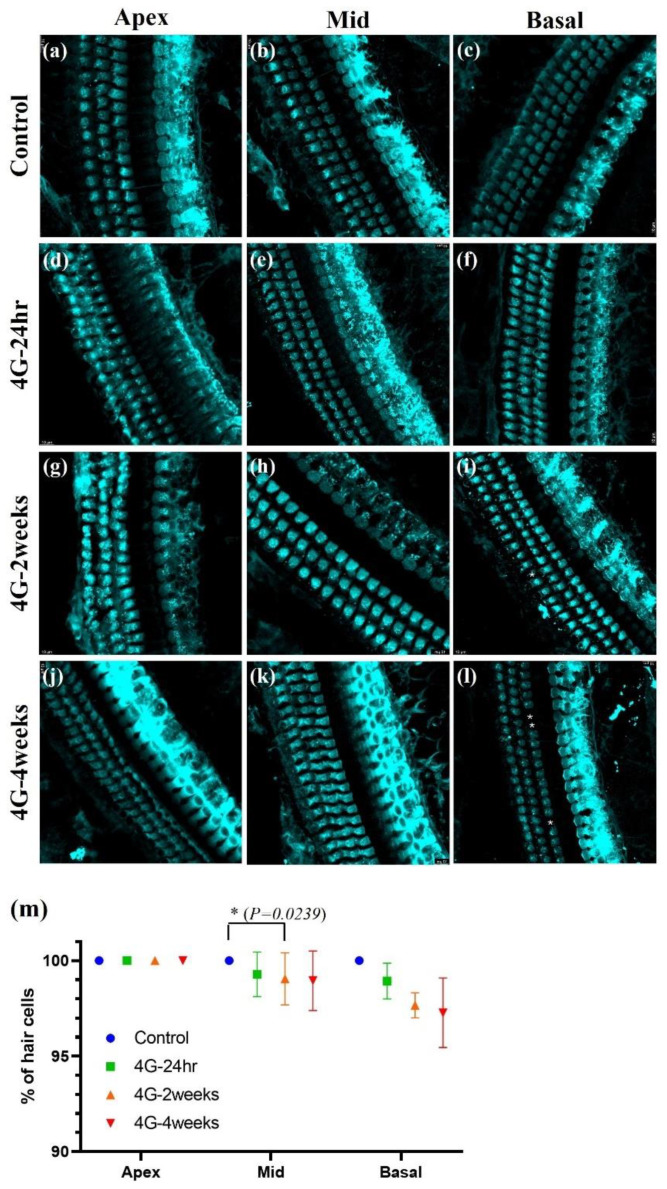
Image of hair cells exposed to hypergravity (4G). Whole-mount preparation showing Myo 7a-positive hair cells in the cochlea. Three rows of outer hair cells and a single row of inner hair cells were present in all groups. (**a**–**c**) Control group: Normal morphology of hair cells. (**d**–**f**) 4G-24hr group. (**g**–**i**) 4G-2weeks group: Loss of OHC was observed during the basal turn. (**j**–**l**) 4G-4weeks group: OHC loss occurred in the middle and basal turns. (**m**) Graph showing the percentage of hair cell survival: In the middle and basal turns hair cell loss was observed in the OHC in the 4G-4weeks group. Scale bar = 10 μm.

We performed immunostaining of whole-mount cochlear tissue using a Myo 7a antibody to visualize and assess cochlear hair cells. Samples were examined from three regions: apex, middle, and basal turns of the cochlea (Figure 4).

In the control group, we observed a typical cochlear structure: three rows of outer hair cells (OHCs) and a single row of inner hair cells (IHCs), all intact and without any apparent loss (Figure 4a–c). This normal morphology was consistent across all cochlear turns. However, in the groups exposed to 4G hypergravity for 2 and 4 weeks, we observed notable changes in hair cell morphology, particularly in OHCs (Figure 4g–l). The most striking difference was the loss of OHCs, which was evident in both the 2-week and 4-week exposure groups. Interestingly, hair cell loss showed a gradient effect along the cochlear turns (Figure 4m). The most pronounced loss was observed in the basal turn, which was responsible for the detection of high-frequency sounds. This suggests that high-frequency hearing is more susceptible to damage from prolonged exposure to hypergravity. These findings indicate that extended exposure to hypergravity can lead to significant structural changes in the cochlea, particularly affecting the outer hair cells. The preferential loss of hair cells during the basal turn suggests that high-frequency hearing is more vulnerable to hypergravity-induced damage.

### 2.3. Neurofilament Analysis

We used NF 200, a characterized neural marker for both type I and type II Spiral Ganglion Neurons (SGNs), to assess the impact of hypergravity on cochlear neural structures [14]. Confocal imaging of cochlear surface preparations co-stained with NF 200 and Myo 7a allowed us to visualize both auditory nerve fibers (afferent and efferent) and hair cell morphology.

**Figure 5:**
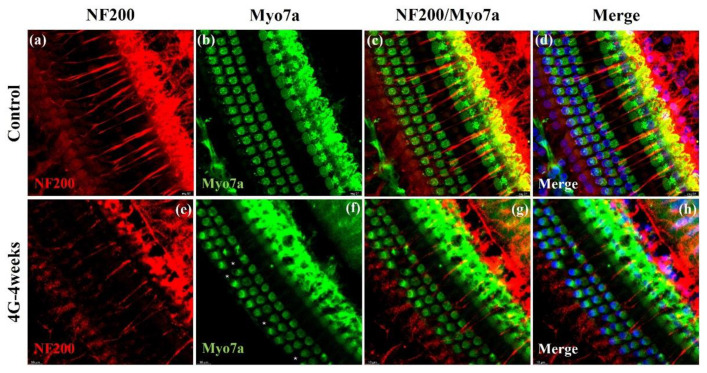
Immuno-staining of cochlear whole-mounts showing the morphology of auditory nerve fibers in the control and 4G-4weeks group. Changes in the auditory nerve fibers in cochlear whole-mounts exposed to control and 4G-4weeks conditions. (**a**–**d**) Control group: Normal nerve fibers. (**e**–**h**) 4G-4weeks group: Loss of NF 200^+^ afferent fibers innervating IHCs on the cochlear surface. NF200 (red), Myo VIIa (green), and DAPI (blue) staining. *: loss of hair cells. Scale bar = 10 μm.

Our analysis showed significant changes in the 4G-4weeks group compared to the control group. The NF200-positive fiber density was markedly decreased in the 4G-4weeks group compared to the control group (Figure 5a,e). This reduction suggests the loss or degeneration of auditory nerve fibers following prolonged hypergravity exposure. Hair cells exhibited normal morphology (Figure 5b) with intact structures and organization in the control group. However, in the 4G-4weeks group, consistent with our previous results, a significant loss of OHCs was noted (Figure 5f). These findings provide strong evidence that prolonged exposure to hypergravity conditions (4G-4weeks) induces substantial changes in cochlear neural architecture. The observed reduction in NF200-positive fiber density coupled with the loss of OHCs strongly suggests that hypergravity exposure leads to both neural and sensory cell damage in the inner ear. Collectively, these results indicate that exposure to hypergravity can induce significant structural changes in the cochlea, potentially leading to impaired hearing. The concurrent loss of neural fibers and hair cells underscores the comprehensive impact of hypergravity on the auditory system, affecting both its neural and sensory components.

### 2.4. Analysis of Synapse

**Figure 6:**
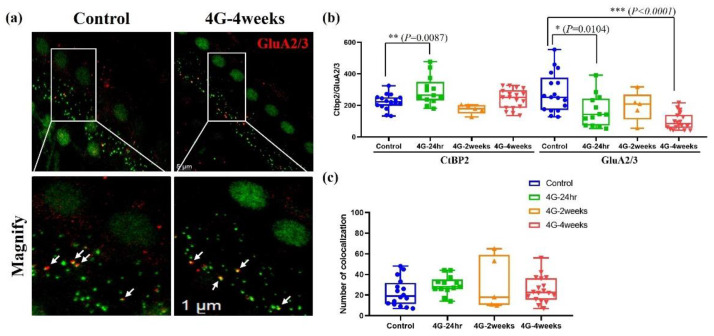
Presynaptic and postsynaptic marker counts in the control and 4G-4weeks group. Synapses between spiral ganglion neurons (SGNs) and IHCs were quantified in organs of Corti isolated from the control and 4G-4weeks group. (**a**) Presynaptic (CtBP2) and postsynaptic (GluA2/3) markers were observed in the control group and 4G-4weeks group through cochlear surface preparation (white arrow). Images, presented as z-projections, were generated using stacks of confocal micrographs for each group. (**b**) The number of presynapses and postsynapses in each group was quantified. (**c**) Analyses of the co-localization of pre- and postsynaptic markers were similar in all groups. GluA2/3 (red) and CtBP2 (green).

The strategy for imaging whole-mounted cochlea is illustrated in Figure 6, which compares a control group with the 4G-4 week group stained using a presynaptic marker (anti-CtBP2, green) and a postsynaptic marker (anti-GluA2/3, red). The distribution of synapses in the inner hair cells of the cochlea was quantified using immunohistochemical image analysis (Figure 6). Compared to the control group, the presynaptic ribbon showed a temporary increase in the hair cells of the group exposed to hypergravity stimulation for 24 h. However, this increase returned to baseline levels with continued exposure for 2 weeks and 4 weeks. Conversely, a gradient decrease in postsynaptic density was observed with prolonged 4G exposure (Figure 6b). Additionally, no significant differences were detected in the co-localization of presynaptic ribbons and postsynaptic receptors (Figure 6c). These findings highlight the capacity of hair cells to exhibit synaptic plasticity when adapting to persistent gravitational environments. These results align closely with reports of decreased hearing function observed in astronauts after returning from spaceflight, emphasizing the potential impacts of prolonged gravitational changes on auditory synapses.

## 3. Discussion

Previous studies have extensively investigated structural changes in the vestibular organ and alterations in utricular hair cell synapse density [6,15,16]. Space sickness typically occurs at the onset of spaceflight and transitions into an adaptation phase with continued exposure to stimulation [17,18]. This condition arises from mismatched signals between different sensory organs [4,7]. When space sickness occurs, astronauts may be unable to perform tasks effectively, making it a significant challenge in space medicine [19,20]. Due to problems such as reduced quality of life and the inability to carry out daily life activities resulting from space motion sickness, many researchers are conducting studies to better understand the mechanisms of space motion sickness and the role of the vestibular system [4,18,21]. However, the cochlea, despite its structural and functional similarities to vestibular organs and close anatomical proximity, remains largely unexplored. It is reasonable to infer that if space stimulation significantly affects the vestibular system to cause motion sickness, a similar impact may occur in the auditory system.

Interestingly, 100% of astronauts experience temporary hearing threshold shifts during spaceflight, and 81% report permanent hearing loss [22]. Hearing loss can severely affect quality of life, leading to depression and social isolation, placing a burden on the patient and those around them [23,24].

This study comprehensively investigated the effects of hypergravitational stimulation on the auditory system. Using an animal model exposed to a 4G hypergravity environment for 24 h, 2 weeks, and 4 weeks, hearing thresholds, hair cell loss, changes in neural structure, and changes in synapse density were evaluated to demonstrate various aspects of hearing impairment. First, the auditory brainstem response (ABR) analysis showed that hearing thresholds increased with prolonged hypergravity exposure. After 24 h, 2 weeks, and 4 weeks of exposure, changes in auditory function did not exhibit a dramatic, sequential pattern. However, a more pronounced and discernible alteration in hearing was observed as the duration of exposure increased. Persistent changes in amplitude and latency were observed even in the 2-week group, indicating potential long-term effects. Notably, the 4-week group exhibited irreversible hearing damage, suggesting that prolonged exposure to hypergravity may lead to permanent auditory impairment. The observed time-dependent damage to hair cells highlights the necessity for further investigation to clarify the underlying mechanisms and long-term impacts of prolonged hypergravity exposure on auditory function. In particular, hearing loss was observed in the high-frequency region, suggesting heightened vulnerability of high-frequency hearing to hypergravity. This phenomenon is similar to hearing loss caused by aging and oxidative stress [25,26,27,28,29,30]. OHCs were more sensitive than IHCs, particularly in high-frequency regions [31]. These results align with reports of hearing loss among astronauts upon their return to Earth and provide important insights into the long-term effects of hypergravity on the auditory system. Second, the loss of OHCs was observed using immunofluorescence analysis. In particular, the significant loss in the basal turn demonstrates that hypergravity stimulation can have direct effects on inner ear structures. This result is consistent with high-frequency hearing loss, suggesting that high-frequency areas should be considered when establishing hearing protection strategies in hypergravity. In conclusion, we found that long-term exposure to hypergravity stimulation deteriorates hearing function, a significant result that demonstrates the functional and structural changes in the inner ear caused by a single gravity stimulation environment. Third, neurofilament (NF 200) analysis showed that hypergravity stimulation affects auditory nerve structures. A decrease in NF200-positive fiber density suggests loss or degeneration of auditory nerve fibers, which is an important finding for understanding the neurological mechanisms of hearing loss. Lastly, synaptic density analysis demonstrated the inner ear adaptation mechanism to hypergravity. The initially observed transient increase in presynaptic ribbons, followed by recovery, can be viewed as an immediate compensatory mechanism of the nervous system to adapt to new environments. We propose that in response to 4G hypergravity stimulation for 24 h, the cochlear presynapse initially hyper-reacted and increased the number of synapses. In contrast, the postsynaptic density gradually decreased. This can be attributed to several factors. First, the damage was caused by constant stress. As the hypergravity environment persists, accumulated stress may occur in the postsynaptic structures, resulting in the progressive loss of synapses [15]. Second, there is an imbalance in neurotransmitters. Excessive early activation of the presynapse can lead to neurotransmitter imbalances, which can cause long-term damage to postsynaptic structures [32]. Finally, there are changes in the energy metabolism. Sustained hypergravity may impair cochlear energy metabolism, leading to problems with the energy supply required to maintain postsynaptic structures [33]. This phenomenon demonstrates the complex impact of hypergravity on the auditory system and suggests different adaptation mechanisms at the pre- and postsynapses. Taken together, these findings provide important clues for understanding how a long-term stay in an altered gravity environment affects the auditory system.

Interestingly, despite differences in presynaptic and postsynaptic responses to hypergravity stimulation, their colocalization remained unchanged. This phenomenon can be explained by neurotransmitter maintenance mechanisms [34,35,36,37]. First, synaptic plasticity must remain balanced. Despite individual changes in presynaptic and postsynaptic structures, homeostatic mechanisms in the nervous system likely maintain overall synaptic ability [37]. This may be an adaptive response that maintains the functional stability of neural circuits. Second, functional compensation may occur. Quantitative changes that occur in presynaptic and postsynaptic structures could offset each other, thereby maintaining functionally significant colocalization [38]. Finally, balance between synapse formation and elimination [39,40] might sustain colocalization. During the initial increase in presynapses and the continued decrease in postsynapses, the formation of new synapses and the elimination of existing synapses may have been balanced to maintain overall colocalization. These phenomena reflect the complexity of neuroplastic mechanisms [41]. Hypergravity stimulation increases activity in tissues related to the vestibular system, which can be interpreted as adaptive changes in the nervous system.

These findings imply that hearing protection in hypergravity environments must extend beyond mere sound isolation to encompass comprehensive strategies for safeguarding the inner ear’s structure and function. Future research is needed to explore the reversibility and long-term effects of these changes, as well as possible prevention and treatment strategies. Although prolonged exposure to hypergravity is uncommon in daily life, specific specialized contexts, such as space exploration, can impose altered gravitational forces on individuals. The potential impact of gravitational changes on human physiology, particularly inner ear function, necessitates further research into their effects on the vestibular and auditory systems. Such studies could be crucial for advancing space medicine and understanding human adaptation to extreme gravitational environments.

While our study provides valuable insights, its generalizability could be improved by increasing the sample size and including a more diverse population (e.g., different ages and genders). Additionally, future studies could examine the chronic effects over periods of 4 weeks or longer and compare the effects of hypergravity and microgravity [42]. Additionally, research is needed to elucidate the exact molecular and cellular pathways involved in hypergravity-induced hearing impairment. Finally, changes in auditory function caused by other space-related stressors (e.g., radiation, sleep deprivation, and isolation) need to be studied [43]. Addressing these limitations and expanding future research will contribute to a comprehensive understanding of the effects of gravity on the auditory system.

## 4. Materials and Methods

### 4.1. Animals

Male Sprague–Dawley (SD) rats, aged 6 weeks and weighing 250–300 g, were used in this study. The rats were purchased from Orient Bio Inc. (Seongnam, Republic of Korea). All experimental protocols were approved by the Institutional Animal Care and Use Committee of Inha University (INHA 240610-937). All methods were conducted in accordance with relevant guidelines and regulations.

### 4.2. Hypergravity Stimulation

**Figure 7:**
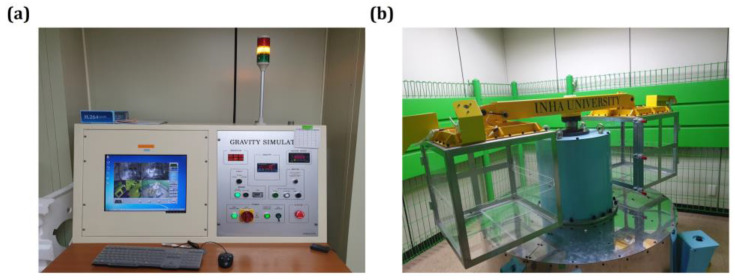
Hypergravity device. We constructed a hypergravity device to establish a gravity stimulation animal model. (**a**) Hypergravity controller. Gravity stimulation can be controlled with an external controller, and a camera is installed to observe the animal’s condition while gravity stimulation is applied. Through cameras installed on each arm, the interior can be observed in real-time while exposed to hypergravity stimulation. (**b**) Hypergravity device. The hypergravity device has two rotatory arms, and each arm can accommodate four cages.

Hypergravity conditions were created using a centrifuge equipped with two rotatory arms that accommodated cages (Figure 7, Supplementary Video S1). The animals were exposed to 4G hypergravity and allowed a 1 h rest period at the same time daily, during which they received adequate food and water. Hypergravity exposure durations were 24 h, 2 weeks, and 4 weeks, with experiments performed at the end of each stimulation period to assess the effects.

### 4.3. Auditory Brainstem Response (ABR) Test

ABR recordings were conducted simultaneously at the endpoints of hypergravity exposure (control, 24 h, 2-week, and 4-week groups; 10 rats per group). Rats were anesthetized with ketamine (100 mg/kg; Yuhan Corporation, Seoul, Republic of Korea) and xylazine (1 mg/kg; Rumpun, Korea Elanco, Seoul, Republic of Korea) before being placed in a sound-attenuated chamber. Body temperature was maintained at 37 °C using a heating pad. Ground and recording electrodes were placed subcutaneously on the scalp, and a calibrated transducer (Tucker Davis, Alachua, FL, USA) was placed in the right pinna.

The clicks and pure-tone pips at 4, 8, 16, 24, and 32 kHz were presented at 10 dB to 90 dB sound pressure levels (SPL; rms for click stimuli) in 10 dB increments. After click and pure-tone recordings, the amplitude (nV) and latency (ms) were analyzed in wave 1 and wave 2.

### 4.4. Immunostaining Assay

After ABR recordings, all animals were euthanized, and their cochleae were dissected from the temporal bone and fixed in 10% paraformaldehyde in phosphate-buffered saline (PBS) overnight on a rocker at 4 °C. The samples were decalcified in 0.25 M ethylenediaminetetraacetic acid (EDTA) solution for up to 21 days (duration was age-dependent), with daily solution changes. Decalcified cochleae were micro-dissected at the apex, middle, and basal turns, and the tectorial and Reisner’s membranes were removed. Each sample was permeabilized in 0.05% Triton X-100 in PBS for 30 min and then in a blocking solution containing 10% bovine serum albumin (BSA) for 60 min. The samples were incubated with the primary antibody overnight at 4 °C. The washed tissues were then incubated with a fluorescent dye-conjugated secondary antibody for 4 h at room temperature. The following primary and secondary antibodies were used: MyoVIIa (Santa Cruz Biotechnology, Inc., Dallas, TX, USA), anti-CtBP2 (BD Biosciences, San Jose, CA, USA), anti-Glutamate Receptor 2 and 3 (Merck Millipore, Burlington, VT, USA), NF200 (Proteintech, Rosemont, IL, USA), Alexa Fluor^TM^ 488 Goat anti-Mouse IgG1 (Thermo Fisher, a-21121, Waltham, MA, USA), Alexa Fluor^TM^ 546 Goat anti-Mouse IgG2a (Thermo, a-21133, Waltham, MA, USA), Alexa Fluor^TM^ 647 Goat anti-Rabbit (Thermo, a-21245). Images were captured with Leica Stellaris 5 confocal microscopes (Wetzlar, Germany).

### 4.5. Statistical Analysis

Statistical analyses were performed using GraphPad Prism (Version 8.0.1, GraphPad Software, San Diego, CA, USA). A paired *t*-test was used to compare thresholds between the control and hypergravity groups (4G-24hr, 4G-2week, and 4G-4week). Statistical significance was defined as * *p* < 0.05, ** *p* < 0.01, and *** *p* < 0.001.

## 5. Conclusions

This study aimed to investigate the functional and morphological changes in the inner ear following hypergravity stimulation. The results indicated a significant hearing threshold shift and latency delay, as well as observable hair cell loss and alterations in nerve fiber density after hypergravity exposure. These findings suggest that hypergravity can induce substantial changes in the inner ear. Although this study was limited to hypergravity stimulation, it contributes valuable insights into the mechanisms of hearing impairment. To enhance the generalizability of these findings, future studies should consider increasing the sample size and incorporating a more diverse population, including various ages and genders. This approach will be in developing a more comprehensive understanding of the effects of hypergravity on the auditory system.

## Data Availability

Data is contained within the article and Supplementary Materials.

## Supplementary Materials

The following supporting information can be downloaded at: https://www.mdpi.com/article/10.3390/ijms26020758/s1.

## Abbreviations

The following abbreviations are used in this manuscript:

ABR	Auditory brainstem response
BSA	Bovine serum albumin
EDTA	Ethylenediaminetetraacetic acid
IHC	Inner hair cell
NF	Neurofilament
OHC	Outer hair cell
PBS	Phosphate-buffered saline
SD	Sprague–Dawley
SGN	Spiral ganglion neuron
